# Social resistance drives persistent transmission of Ebola virus disease in Eastern Democratic Republic of Congo: A mixed-methods study

**DOI:** 10.1371/journal.pone.0223104

**Published:** 2019-09-26

**Authors:** Kasereka Masumbuko Claude, Jack Underschultz, Michael T. Hawkes

**Affiliations:** 1 Department of Medicine, Université Catholique de Graben, Butembo, Democratic Republic of Congo; 2 Department of Pediatrics, University of Alberta, Edmonton, Canada; 3 Department of Medical Microbiology and Immunology, University of Alberta, Edmonton, Canada; 4 Department of Global Health, School of Public Health, University of Alberta, Edmonton, Canada; Tulane University, UNITED STATES

## Abstract

**Background:**

The second largest Ebola virus disease (EVD) epidemic in history is currently raging in Eastern Democratic Republic of Congo (DRC). Stubbornly persistent EVD transmission has been associated with social resistance, ranging from passive non-compliance to overt acts of aggression toward EVD reponse teams.

**Methods:**

We explored community resistance using focus group discussions and assessed the prevalence of resistant views using standardized questionnaires.

**Results:**

Despite being generally cooperative and appreciative of the EVD response (led by the government of DRC with support from the international community), focus group participants provided eyewitness accounts of aggressive resistance to control efforts, consistent with recent media reports. Mistrust of EVD response teams was fueled by perceived inadequacies of the response effort (“herd medicine”), suspicion of mercenary motives, and violation of cultural burial mores (“makeshift plastic morgue”). Survey questionnaires found that the majority of respondents had compliant attitudes with respect to EVD control. Nonetheless, 78/630 (12%) respondents believed that EVD was fabricated and did not exist in the area, 482/630 (72%) were dissatisfied with or mistrustful of the EVD response, and 60/630 (9%) sympathized with perpetrators of overt hostility. Furthermore, 102/630 (15%) expressed non-compliant intentions in the case of EVD illness or death in a family member, including hiding from the health authorities, touching the body, or refusing to welcome an official burial team. Denial of the biomedical discourse and dissatisfaction/mistrust of the EVD response were statistically significantly associated with indicators of social resistance.

**Conclusions:**

We concluded that **s**ocial resistance to EVD control efforts was prevalent among focus group and survey participants. Mistrust, with deep political and historical roots in this area besieged by chronic violence and neglected by the outside world, may fuel social resistance. Resistant attitudes may be refractory to short-lived community engagement efforts targeting the epidemic but not the broader humanitarian crisis in Eastern DRC.

## Introduction

Ebola virus disease (EVD) is a lethal, infectious hemorrhagic fever that occurs in outbreaks in equatorial Africa [1]. The 2014–2016 West Africa epidemic was the largest to date, claiming the lives of over 11,000 people [2]. The second largest outbreak in history currently spreads through eastern Democratic Republic of the Congo (DRC) since it began on 1 August 2018, in the North Kivu Province. Unlike past successfully contained outbreaks in the DRC, the current outbreak is relentless, despite intensive efforts toward case detection, contact tracing and management, safe and dignified burials, and ring vaccination. As of 10 July 2019, there have been a total of 2357 confirmed cases and 1553 confirmed deaths [3].

The ongoing EVD epidemic has prompted questions as to what may be driving the persistent transmission. Active conflict, high population density, large numbers of internally displaced persons (IDPs), and mass migrations have been identified as promoting factors [3]. In addition, community attitudes and behaviours may also contribute, as in past persistent EVD epidemics [4–6]. During the 2014–16 outbreak in Liberia, citizens who distrusted their government were less compliant with EVD control policies [7]. Mistrust may also extend to foreign response teams, and “social resistance,” ranging from passive non-compliance to overt acts of violence, has been witnessed during past EVD outbreaks [8]. In Guinea, the 2014–2016 epidemic was complicated by riots, stoning of vehicles, and even deaths of outreach workers [8]. The factors that promoted social resistance included low level of care in EVD treatment centers (ETCs), lack of a traditional burial for the deceased, and a distrust of foreigners “profiting” from the outbreak [8–10]. Past studies during other EVD epidemics suggest that root causes of resistance or reticence relate to five domains: (1) rumours; (2) fear; (3) mistrust and lack of confidence in the authorities; (4) denial of the biomedical discourse; and (5) desire to be autonomous and avoid exogenous contamination [8, 11].

The objective of this study was to explore social resistance to EVD control efforts during the current persistent outbreak in Eastern DRC. We collected qualitative data through focus group discussions (FGDs), and quantitative data via survery questionnaire, sampling the active EVD transmission region of Butembo and its surrounding communities. Findings from this mixed-methods study may inform public health messaging and control efforts in this challenging epidemic.

## Methods

### Design

We conducted a mixed-methods study with qualitative (FGDs) and quantitative (18 item survey questionnaire) data collection. Mixed-methods research seeks to integrate and triangulate findings from qualitative and quantitative methods. Its pragmatic focus contrasts with methodologic purism [12]. Compared to monomethod studies, convergence of findings stemming from two or more methods may enhance the belief that the results are valid (multiple operationalism) [13]. Previous authors have highlighted the value of such real-time data collection that examines real-time emergent trends and integrates community attitudes, behaviors, and responses into epidemiological research [14].

### Study setting and participants

Even prior to the EVD outbreak, the DRC has been the stage of a complex humanitarian crisis that has lasted decades and is characterized by mortality rates 70% higher than pre-war levels, and 55% greater than neighboring sub-Saharan African countries [15]. Violent conflict has displaced over one million people in the densely populated North Kivu and Ituri provinces in Eastern DRC resulting in internally displaced persons (IDPs) residing in temporary camps. The provinces share porous borders with Uganda and Rwanda so there is frequent emigration of refugees to neighbouring countries. Political instability, exacerbated by a long-delayed presidential election in December 2018, has contributed to an atmosphere of lawlessness and impunity in the Eastern regions of the country, long neglected by the national government and the international community [16].

Participants of FGDs and survey questionnaires were purposively selected (convenience sample) from the geographic regions affected by the EVD epidemic. Key informants included nurses and clinicians, community members in the cities/villages of Béni, Butembo, and Mangina, as well as IDPs in the Komanda camp. Each FGD had a mixed composition of these key informants. FGDs were conducted in the fourth month of the epidemic, during a peak in EVD transmission. Participants were 18 years of age or older.

### Focus group discussions

FGDs were conducted in the local languages (Kinande, Kiswahili, and French) by one of the study authors experienced in FGD methodology (KMC). FGDs included 5 participants in each group and lasted approximately 45 minutes each [17]. Participants were not remunerated. Discussions were recorded, then translated and transcribed by KMC into English for subsequent analysis. FGD questions were elastic, open-ended, and probing, allowing participants to shape the discussion. Questions were adapted from one FGD to the next allowing an iterative approach to confirm findings and explore emerging themes in greater depth. FGDs were continued until a point of saturation, when no new themes emerged in discussions [18]. Thematic analysis was used to identify, analyze, and report themes in the discussions [19]. Two investigators (JU and MH) read the transcripts several times, noted preliminary ideas, produced initial codes, then generated and refined themes. Microsoft Word was used for qualitative data transcription and thematic analysis. Representative quotations as well as statements of particular interest were extracted to support the themes. Where language was potentially identifying (e.g., names of towns and organizations), these were redacted. Themes that emerged from the FGDs were used to inform quantitative survey content.

### Survey questionnaire

An 18-item questionnaire was developed, based on past questionnaires used in Guinea and the DRC [5, 20] and informed by qualitative results from FGDs. In addition to participant demographics, questions focused on several key domains relevant to social resistance to EVD control efforts:

1. **Perceived risk of EVD.** Participants were asked to identify whether they felt they were at high, intermediate or low risk of contracting EVD, as in a previous EVD survey [5].
2. **Denial of biomedical discourse.** As a possible upstream cause of social resistance [8], we assessed respondents’ agreement with the statement: “EVD is a fabrication, the disease does not exist here.” Participants were asked to rate their agreement on a Likert scale from 1 to 5 (1: “strongly agree”; 2: “agree”; 3: “neutral”; 4: “disagree”; and 5: “strongly disagree”). Denial (binary variable) was defined as a response of “strongly agree” or “agree,” and no denial was defined as a response of “neutral,” “disagree,” or “strongly disagree.”
3. **Dissatisfaction and mistrust of the EVD response team.** In FGDs, some participants expressed viewpoints that were critical or mistrustful of the EVD response, which may represent root causes of social resistance [8, 11]. To assess the prevalence of these views, statements were generated that were representative of some qualitative findings, and respondents were asked to rate their agreement on a 5-point Likert scale. We used five statements to gauge mistrust and lack of confidence in the EVD control efforts: “I am not satisfied with the EVD response in Eastern DRC,” “The EVD response is not well organized,” “The foreigners come to assist with EVD control have a poor understanding of the local culture and regional situation,” “I don’t trust the foreigners come to assist with EVD control,” and “The foreigners have come to make a profit from this epidemic.” Correlation among the five items was assessed using a matrix of Spearman’s rank correlation coefficients and associated p-values. Dissatisfaction/mistrust (binary variable) was defined as agreement or strong agreement with one or more of these five items.
4. **Vaccine acceptability and community engagement.** Some FGD participants felt that ring vaccination as a public health strategy was inadequate, which may have undercut confidence in the response effort. We assessed attitudes toward universal *versus* targeted vaccination using the statement “We should vaccinate everybody against EVD, not only the contacts.” (5-point Likert scale). Lack of engagement of local medical staff and community members was another critique that arose from FGDs. We assessed agreement with the statement “The epidemic will continue as long as the local community is not engaged in control efforts” (5-point Likert scale).
5. **Support for incidents of overt hostility.** Beyond non-compliance with public health recommendations, the current outbreak in Eastern DRC has witnessed acts of aggression toward health facilities and EVD response teams [21]. As a marker of sympathy or support for more extreme social resistance to control efforts, we evaluated respondents’ agreement with the statement “People are justified in resisting the EVD control efforts (e.g., throwing stones, destruction of hospital property, taking away infected bodies, or chasing away the vaccination team)” (5-point Likert scale). With respect to this putative index of social resistance, participants were classified as “resistant-support hostility” if they agreed or strongly agreed with this statement and were classified as “compliant” otherwise.
6. **Intentions to comply with control efforts in case of EVD illness or death in family.** We included four items asking about personal intentions related to EVD control, based on a past study [5]: would not bring infected person to the treatment centre, would hide person from health authorities, would touch body of deceased, or would refuse that an official burial team take care of the body. Correlation between the four items was assessed using a matrix of Spearman’s rank correlation coefficients and associated p-values. With respect to this second putative index of social resistance, respondents were classified as “resistant-non-compliant” if they expressed one or more of the four resistant intentions, and “compliant” if they expressed none of these intentions.

Survey respondents represented a convenience sample of community members and were recruited as follows. Surveyors were chosen from leaders among the medical students at the Université Catholique du Graben, Butembo, DRC. Medical students are trusted members of the local community with strong literacy skills, biomedical understanding of EVD, and tacit cultural competencies. They used their social networks (including faith-based assemblies and market gatherings) to recruit survey participants. The survey was written in French and was completed by the participants themselves when they could read and write in French. For those participants who could not read and write in French, the standardized questionnaire was completed by surveyors using verbal interviews of participants, in their preferred language (Kiswahili, Kinande, and/or French). For data collection, we used paper forms; for data entry and management, we used an electronic tool, KoBoToolbox [22].

### Statistical analysis

GraphPad Prism version 6 (GraphPad Software Inc., La Jolla, CA, USA, 2012), and R (version 3.3.3, R core team, 2017) were used for data analyses. Descriptive statistics used proportions or median and interquartile range (IQR) for dichotomous and continuous variables, respectively. Correlations between questionnaire items using a 5-point Likert scale were analysed by the non-parametric Spearman’s rank correlation coefficient (ρ) and the associated p-value, testing the hypothesis that the correlation coefficient was different from zero. We hypothesized that denial and/or dissatisfaction/mistrust would be associated with social resistance, measured by two dichotomous indices of resistance: support for overt hostility and non-compliant intentions. For predictor variables, we examined demographic characteristics (age, sex, occupation, and education level) and attitudes (risk perception, denial, and dissatisfaction/mistrust). We used two-by-two contingency tables to test for an association between the predictor variables and indices of social resistance, quantifying the strength of the association using the odds ratio [23] and its 95% confidence interval (CI), and using the Chi-squared or Fisher’s exact statistical tests, as appropriate. The odds ratio is computed as the cross-product ratio of the entries in the 2-by-2 contingency table of two binary variables [23]. To adjust for potential confounding effects between variables, we constructed multivariable logistic regression models based on the bivarable analysis. Variables were included in the multivariable model if the p-value from the bivariable association was <0.1. Model results were expressed as adjusted odds ratios with 95% CIs and associated p-values.

### Ethics approval

The study was approved by the Comité d’Éthique du Nord Kivu (Centre Hospitalier Universitaire du Graben, Butembo, DRC). Oral consent was obtained from all participants. The ethics committee waived the need for written informed consent for the following reasons: (1) survey questionnaires and FGDs carried minimal risk to participants; (2) participation in the survey/FGDs constituted tacit consent; and (3) excessive participant contact, including signing paperwork, was discouraged in the context of an EVD outbreak with potential for viral transmission by direct person-to-person contact and/or by fomites.

## Results

### Focus group discussions

We began with a qualitative exploration of social resistance to EVD control efforts. We conducted four FGDs, involving 20 participants (total); participant characteristics are shown in Table 1. All participants were local residents of Eastern DRC; no foreign nationals or international members of the EVD response were included. FGDs produced rich qualitative data, from which we derived the following themes: (1) reports of compliance with and resistance to EVD response team; (2) poor quality, insufficiency, and cultural incongruity of EVD response; (3) lack of engagement of local personnel; and (4) mistrust of EVD response team. We elaborate on each theme, below, supported by representative quotations. For all quotations below (translated to English from original French), we include the original verbatim French quotations in Table A in S1 File.

**Table 1:** Description of focus group discussion (FGD) composition.

Focus group discussion (FGD)	Unique identifier
**FGD#1 (5 participants)**	
Doctor at regular HCF	ID#01
Nun and health centre nurse at regular HCF	ID#02
Community Health Worker affiliated with regular HCF	ID#03
Community member from neighborhood around HCF	ID#04
Community member from neighborhood around HCF	ID#05
**FGD#2 (5 participants)**	
Nurse anesthetist at regular HCF	ID#06
Member of NGO participating in EVD response	ID#07
Community Health Worker affiliated with regular HCF	ID#08
Community member from neighborhood around HCF	ID#09
IDP camp resident	ID#10
**FGD#3 (5 participants)**	
Doctor at regular HCF	ID#11
Member of NGO participating in EVD response	ID#12
Community Health Worker affiliated with regular HCF	ID#13
Community member from neighborhood around HCF	ID#14
Morgue worker at regular HCF	ID#15
**FGD#4 (5 participants)**	
Doctor at regular HCF	ID#16
Doctor, member of EVD vaccine veam	ID#17
Doctor, member of EVD case management team	ID#18
Nun and head nurse at regular HCF	ID#19
Nurse and Ebola survivor, worked in ETU	ID#20

### Compliance with and resistance to EVD response team

According to FGD participants, many people welcomed epidemic control measures, sought assistance, and appreciated national and international aid:

“The epidemic is lethal and the eruption is strong, without the participation of the outside the sickness would have already taken another dimension and even crossed certain borders of neighboring countries. We therefore say thank you to all those who are supporting us.” (doctor, ID#11)

However, participants also provided accounts of violent backlash against medical establishments or teams:

“A few days ago, a health center was attacked by the angry community and two police officers were left with injuries due to throwing stones. There were gunshots and property damage.” (doctor, ID#16)

“The community is having a hard time digesting the high number of deaths at the ETC [EVD treatment centre]. The ETC is well guarded, so the community attacked the health post that transferred the patient to the ETC.” (doctor, ID#16)

### Poor quality, insufficiency, and cultural incongruity of EVD response

According to FGD pariticpants, the EVD response in Butembo was characterized by a highly visible, apparently well-financed, but poorly trusted foreign element, including both foreign Congolese nationals (from other areas of the DRC) and international workers. One FGD participant emphasized the importance of local trusted sources of information and leadership over formal media (e.g., radio) by outsiders:

“Everyone has a leader that he has confidence in. These *étrangers au milieu* [strangers in our midst] because of money didn’t take this aspect into consideration.” (doctor, ID#17)

“The training should have been done *de bouche à l’oreille* [from mouth to the ear] in an informal manner.” (doctor, ID#18)

The foreign effort was seen as ineffectual in some instances:

“A Béninois will do his messaging in French, a *Kinois* [person from country capital Kinshasa] in Lingala. And that, what impact do you think it will have?” (doctor, ID#17, note that the locally spoken languages are Kiswahili and Kinande, not French or Lingala)

The handling of corpses (i.e., safe and dignified burials) was viewed as alien and was a source of dissatisfaction reported by FGD participants, and cited as a reason for community resistance to control efforts:

“It’s also a problem of culture. No, really, the way of handling the body is horrible. Our culture doesn’t tolerate this. The body is considered like a thing, an object.” (nurse and nun, ID#19)

“The body… is wrapped in a tarp. No one from the family sees the body. Our culture demands that we say goodbye to the one that goes ahead of us to the beyond. It’s a powerful moment for us.” (nurse and nun, ID#19)

“*une morgue de fortune en caoutchouc* [a makeshift plastic morgue].” (doctor, ID#18, describing the way bodies are wrapped in tarpaulin sheets for safe burial)

Separation from the sick relative, possibly indefinite, was not easily tolerated. Moreover, seeing the patient before and after death was important to family members:

“A loved one falls ill, he goes to the ETC, that’s it. You will never see him again or better let’s say it’s a separation without possibility of seeing each other again.” (nurse and nun, ID#19)

“A funeral without saying goodbye to the deceased!! That is a curse, that is.”

(community member, ID#14)

FGD participants cited poor quality of care in the ETC as a reason for failing to present to a treatment centre when EVD is suspected

Inadequacies of the EVD response were frequent explanations for community and local health worker dissatisfaction:

“The medications are not sufficient. We are still scared because the process is slow for these visitors here to be able to believe in what you are telling them. They don’t believe people.” (community member, ID#05)

“Everyone wants [the vaccine]. And according to the criteria, only those who were in contact with the suspected cases. And it’s a procedure to verify the veracity of the claims of someone who is asking for the vaccine.” (doctor, ID#01)

“We informed the health authorities of [city]… but no one answered the phone. Finally, having informed the team, they told us that they would come the next day. Because of the security risk, no travel at night. The body stayed at the health centre without an equipped morgue.” (doctor, ID#11)

Protocolized medicine by foreign teams was seen as callous and impersonal:

“[NGO] is doing *médecine de troupeau* [herd medicine]. When a sheep is sick, well, you have to treat all the sheep in the same way. It’s not individualized medicine on a case-by-case basis, no.” (doctor, ID#18)

“[NGO] has a *protocole figé* [rigid frozen protocol]. EVD patient equals protocol…” (doctor, ID#18)

“For [NGO], the objective is to limit the epidemic, isolate the sick. Even if he dies, no problem, so long as he doesn’t contaminate others.” (doctor, ID#17)

### Lack of engagement of local personnel

EVD control efforts were largely staffed by national and international foreign personnel, and there was a perception among FGD participants that little effort was made to engage local medical personnel. Initially keen to be involved in the EVD response, one local health worker felt under-valued and disengaged from the foreign-led control efforts:

“Understanding what is going on is difficult. Me, I quit just a week into it. I was recruited. I arrived in Béni, I didn’t even know whom to speak to. One person says go there, and another says something else. Finally, we start. Now I pay for my own accommodation. I pay for my own food. They tell me, you have to be in [name of town] (30 km away from [name of town] where I was staying) at 6am in the morning. And then in the morning there was no means of transportation. I take a motorcycle, and the evening, the same thing. So look, I left. People are de-motivated… And if the local people disengage from the process, well it will be a serious humanitarian catastrophe.” (doctor, ID#18)

#### Mistrust of EVD response team

Local health workers were suspicious that national EVD staff were motivated by financial gain. Perceived disparities in local and foreign payscales were keenly felt:

“We can read the spirit of the *Kinois* [workers from Kinshasa], which is ‘no EVD, no job.’ The *Kinois* have come in large numbers, they are afraid of the risk and their remuneration is high relative to that of the locals who in addition are sacrificing themselves for the community. The locals are poorly paid, and that’s the reason for a lot of people quitting. Have you ever heard of a *Kinois* who quit? The disparities are striking and we’ve even written a memo to the authorities. If some locals remain, it’s out of patriotism, a volunteer spirit.” (doctor, ID#17)

“…[despite deaths and infections], unfortunately without hazard pay. The government should think about their case.” (doctor, ID#01)

“As for the health personnel without hazard pay, it’s total fear around cases like these.” (doctor, ID#11)

Beyond perceived mercenary motives and inadequacies of care, a troubling suggestion of genocidal intent of the foreign presence arose in several FGDs:

“The message that the politicians are giving is that EVD is a creation come to exterminate the population of [name of town]. People are manipulated and hold to this.” (doctor, ID#18)

In this context, some precautionary policies were misinterpreted, such as withholding a perceived life-saving vaccine from a vulnerable group, pregnant women:

“for example, they are refusing the vaccine to pregnant women, yet we know that pregnant women are always vaccinated to protect the babies. We are asking if this policy is not a way to exterminate us.” (IDP, ID#10)

In contrast to the care provided by outsider teams, stories of heroism by local health workers emerged in FGDs. One healthcare worker, himself infected and admitted to the ETC, recounted the way he cared for other patients, filling perceived gaps in the medical treatment provided under the “protocol”:

“We helped each other out, mutually. Truth be told, there were others sicker than I was, very weakened…We treated the sick in the ETC. Personally, I bathed another patient who had EVD like me. It was a colleague. Emptying his chamberpot. He had no strength. We reconnected the drips of patients who were psychologically overcome who had disconnected theirs. We communicated with our colleagues who were outside by telephone… We told them the medication needs (requests) and they would throw them to us. We helped a lot of sick people in the face of this [NGO] protocol that doesn’t adapt to the case-by-case.” (nurse, ID#20)

### Survey questionnaire

**Fig 1:**
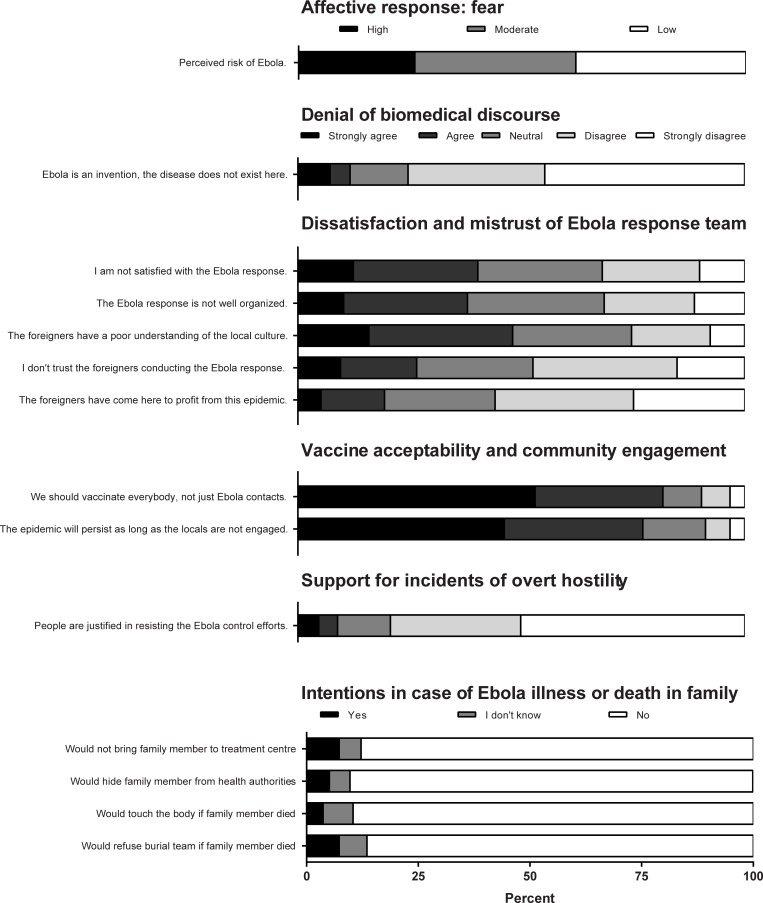
Survey questionnaire responses among 670 participants during EVD outbreak in Eastern DRC. Questions probed social resistance and associated attitudes.

A total of 670 participants were surveyed between 3 and 20 November, 2018. The median age was 23 years (range 18–67) and 298 (43%) were female. Participant characteristics are shown in Table 2. Perceived risk of EVD, attitudes toward the EVD response, and intentions to comply with EVD control measures in case of EVD illness or death in the family are shown in Fig 1.

**Table 2:** Characteristics of survey respondents.

Characteristic	Statistic (N = 670)
**Age [years], median (range)**	23 (18 to 67)
**Sex**	
Male	381 (57%)
Female	289 (43%)
**Employment**	
Student	514 (77%)
Business	46 (7%)
Government/civil service	36 (5%)
Farming	23 (3%)
Doctor, nurse, or healer	16 (2%)
Unemployed	16 (2%)
Other	18 (3%)
**Education level**	
Secondary or higher	632 (94%)
Primary	34 (5%)
None	6 (1%)

Values are n (%) unless otherwise specified

Denial was expressed by 78 (12%) participants and was examined as a possible determinant of social resistance (Table 1). Five questionnaire items related to dissatisfaction/mistrust were statistically significantly correlated (Table B in S1 File). Overall, 482 (72%) respondents agreed or strongly agreed with one or more statements of dissatisfaction/mistrust (Fig 1). Dissatisfaction/mistrust was examined as a possible determinant of social resistance (Table 3).

**Table 3:** Factors associated with resistance *versus* compliance, based on two putative indices of social resistance to EVD control efforts.

*Expressed support for incidents of overt hostility*^*1*^						
	**Resistant-support hostility (N = 60)**	**Compliant (N = 603)**	**OR** **(95% CI)**	**p-value**	**aOR** **(95%CI)**	**p-value**
**Age <25 years** **(ref: age≥25 years)**	17 (28%)	123 (20%)	1.5 (0.8–2.9)	0.2	-	
**Female Sex** **(ref: male)**	30 (50%)	255 (42%)	1.4 (0.77–2.4)	0.31	-	
**Education: secondary or higher** **(ref: primary or no formal education)**	52 (87%)	571 (95%)	0.37 (0.15–0.97)	0.027	0.46 (0.20–1.2)	0.086
**Occupation: student** **(ref: other occupation)**	42 (70%)	468 (78%)	0.67 (0.37–1.3)	0.24		
**High perceived risk of Ebola** **(ref: intermediate or low perceived risk)**	7 (12%)	143 (24%)	0.43 (0.16–0.97)	0.049	0.45 (0.18–0.97)	0.060
**Denial of biomedical discourse** **(ref: no denial of biomedical discourse)**	21 (35%)	56 (9%)	5.2 (2.7–9.9)	<0.0001	3.8 (2.0–7.0)	<0.0001
**Dissatisfaction or mistrust of Ebola response team** **(ref: no dissatisfaction or mistrust of Ebola response team)**	55 (92%)	424 (70%)	4.6 (1.8–15)	0.00075	3.6 (1.5–10)	0.0089
***Non-compliant intention(s) in case of Ebola disease or death in family***^***2***^						
	**Resistant-non-compliant (N = 102)**	**Compliant (N = 563)**	**OR** **(95% CI)**	**p-value**	**aOR** **(95%CI)**	**p-value**
**Age <25 years** **(ref: age≥25 years)**	25 (25%)	117 (21%)	1.2 (0.72–2.1)	0.48		
**Female Sex** **(ref: male)**	49 (48%)	239 (42%)	1.3 (0.8–2)	0.35		
**Education: secondary or above** **(ref: primary or no formal education)**	86 (84%)	539 (96%)	0.24 (0.12–0.5)	<0.0001	0.28 (0.14–0.58)	0.00026
**Occupation: student** **(ref: other occupation)**	73 (72%)	437 (78%)	0.73 (0.44–1.2)	0.23		
**High perceived risk of Ebola** **(ref: intermediate or low perceived risk)**	22 (22%)	129 (23%)	0.93 (0.53–1.6)	0.87		
**Denial of biomedical discourse** **(ref: no denial of biomedical discourse)**	30 (29%)	48 (9%)	4.5 (2.6–7.7)	<0.0001	3.7 (2.1–6.3)	<0.0001
**Dissatisfaction or mistrust of Ebola response team** **(ref: no dissatisfaction or mistrust of Ebola response team)**	86 (85%)	392 (70%)	2.5 (1.4–4.8)	0.0021	1.9 (1.1–3.6)	0.035

Values represent n (%), unless otherwise specified.

A majority of respondents (72%) agreed or strongly agreed with universal (rather than targeted) vaccination. In addition, 77% agreed that community engagement was critical to ending the EVD epidemic. High levels of agreement with these statements were consistent across age and sex strata and were not associated with markers of dissatisfaction or mistrust (p>0.05 for all comparisons).

Sixty (9%) participants expressed support for overt acts of hostility to the EVD response team. Factors associated with this putative indicator of resistance are shown in Table 3 and included lower education level, lower perceived EVD risk, denial, and dissatisfaction/mistrust. Four questionnaire items related to intention to comply with EVD control measures in case of EVD illness or death in a family member, and responses were statistically significantly correlated (Table C in S1 File). Overall, 102 (15%) respondents expressed one or more resistant intentions. Factors associated with this indicator of resistance are shown in Table 3 and included lower education level, denial, and dissatisfaction/mistrust.

## Discussion

Through FGDs and survey questionnaires, we sought to understand social resistance to EVD control efforts, which may explain, at least in part, the stubbornly persistent transmission in Eastern DRC. Our qualitative data support findings from the current [24] and previous EVD epidemics, in which resistance to national and foreign response teams was fueled by perceived inadequacies of the control efforts, attachment to burial rites, suspicion of motives, lack of engagement of local health care personnel, and a legacy of neglect of public health priorities by the state and international agencies [8]. Our survey results suggest that a substantial minority of respondents endorsed views suggestive of “resistance” and that resistance was associated with denial of the biomedical discourse and dissatisfaction/mistrust of the EVD response team (Table 3). In addition, higher perceived risk of EVD was associated with lower support for incidents of overt hostility in a bivariable but not a multivariable analysis (Table 3). For the measurement of dissatisfaction/mistrust, we used several related questionnaire items, which were correlated with each other (Table C in S1 File), suggesting that the questionnaire items were measuring a single unified construct. Likewise, four related questionnaire items pertaining to resistant intentions in case of EVD in a family member were correlated with each other (Table C in S1 File). These findings suggest that our questionnaire survey was internally consistent.

Reticence to comply with early isolation of sick patients, vaccination, and safe burial may provide a window for ongoing EVD transmission. In our survey questionnaire, 15% of participants endorsed one or more non-compliant intentions if one of their family members was ill or died of EVD (not taking person to treatment centre, hiding from authorities, touching body, or refusing official burial team, Fig 1). Expressions of dissatisfaction/mistrust were significantly associated with these reticent attitudes (OR 2.5, 95%CI 1.4–4.8, p = 0.0021). In FGDs, lack of confidence in the EVD response appeared to be driven by perceptions of inadequate patient care, callous adherence to protocol (“herd medicine”), and disrespectful handling of corpses (“makeshift plastic morgue”). Similar findings emerged from qualitative data gathered by other organizations in the area and synthesized by the Social Science in Humanitarian Action Platform [24]. In the 2014–16 West Africa EVD outbreak, health workers lamented the low level of care provided in many ETCs [10]. When communities recognized that infected persons would be isolated in the ETCs but they may not be provided supportive care, they lost trust in government and foreign aid workers and avoided ETCs [9]. Analogous examples exist in which citizen compliance with public health recommendations was compromised by lack of trust in government authorities: vaccine hesitancy linked to government mistrust in the UK [25] and US [26] leading to measles outbreaks; HIV/AIDS in the US, which some Americans believed to be a “man-made weapon of racial warfare” [27]; and refusal of polio vaccine in Nigeria leading to a resurgence of cases in multiple neighboring countries [28]. With respect to vaccination, our own group has previously demonstrated a high level of satisfaction and broad community acceptability of the recombinant vesicular stomatitis virus-Zaire Ebolavirus (rVSV-ZEBOV) vaccine [29]. Any dissatisfaction centred around a preference for universal over ring vaccination and policies not to vaccinate vulnerable groups (e.g., pregnant women) [29].

Another source of animosity in our study was the perception of “disaster capitalism” as a driving motive of the national response team. Based on survey questionnaires, the view that foreign response teams had a profit motive was held by 127/630 (20%) of respondents (Fig 1). The *Kinois* (residents of the distant capital Kinshasa), referred to as “strangers in our midst” by one FGD participant, were assumed to be well-paid, displayed visible wealth (hired vehicles), took minimal risks, rarely got infected, and rarely quit. Delays in responding to potentially infected patients and corpses, together with the sluggish roll-out of interventions such as vaccine were therefore sometimes cynically interpreted as an attempt to draw out the epidemic for personal gain (“no EVD, no job”). For some participants, this created a sharp contrast between local health workers, undervalued and poorly rewarded yet paying a high toll of illness from treating the sick, and the foreign nationals, viewed as mercenaries profiteering from the epidemic. Likewise, in Sierra Leone in 2014–16, “all sorts of interest groups mushroomed overnight to collect cash from the Health Ministry” [30], community volunteers had difficulty making people believe they were not profiting from EVD [8], and control efforts were assumed to be lubricated with “EVD money” [8]. More generally, this phenomenon is reminiscent of “disaster capitalism” [31], describing individuals and groups that find ways to make disasters profitable as a new source of income, prolonging relief efforts for personal gain. As an example of “disaster capitalism,”in the context of an earlier environmental disaster (Hurricane Katrina, New Orleans, USA, 2005), bureaucratic processes alienated victims seeking assistance for destroyed property (quotation from previous study: “you are guilty until proven innocent. You are guilty of lying that you own a home.”) [31]. Similarly, in our study, bureaucratic processes in accessing the vaccine against EVD created dissatisfaction and mistrust (“there’s a procedure to verify the veracity of the claims of someone asking for the vaccine,” “They don’t believe people.”)

A majority (77%) of survey respondents agreed or strongly agreed that the epidemic would persist as long as local communities are not engaged. In FGDs, some local health workers, initially keen to be involved, were quickly disillusioned with a lack of support (logistics, accommodation, transport) and had disengaged from the control efforts. Similarly, in the 2014–16 outbreak, community actors were engaged late in the response, did not receive sufficient support [32], and international aid agencies supplanted rather than supported the local level [33]. Other authors have noted that a cornerstone of effective disaster management is that response should begin and end at the local level [33]. In Guinea, Liberia, and Sierra Leone, when deployment of outsiders proved ineffective due to community mistrust, community health workers who had been providing health services prior to the outbreak had their duties expanded to include EVD-related roles [32]. Increased training and engagment of local health professionals may be important to mitigate community resistance in this and future outbreaks.

Beyond passive non-compliance, FGD participants recounted acts of overt hostility from community members. Corroborating these eyewitness accounts, news reports of community members blocking health workers from taking patients to the ETC [34], infected people escaping from the ETC [35, 36], youth gangs forcibly removing corpses of EVD victims [36, 37], and mass street protests with destruction of administrative buildings [38] have recently emerged from Eastern DRC. Clashes in Béni resulted in temporary halting of case-finding and vaccination, directly impacting critical response efforts [39]. Flashpoints of confrontation included burials, as locals bristled at outsiders violating cultural mores [20], attitudes well represented among our FGD respondents. In our survey questionnaire, 9% of respondents felt that such acts of aggression were justified, suggesting that resistance was entrenched in a substantial minority of the community. How to account for such radical opposition to disease control efforts? Violent resistance to EVD teams was also seen in Guinea, where eight outreach workers were killed in 2014, as well as two policemen in 2015 [8]. Difficulties in community engagement, riots, and stoning of vehicles were regularly documented in WHO situation reports [8]. Political origins of mistrust in Guinea [8] and in Eastern DRC have explanatory power in both contexts. In both countries, corruption scandals and expropriation of mining resources by foreign business interests set the stage for suspicion of the government and of outsiders [8]. The motives of foreigners who arrived on scene to control the EVD threat after decades of neglecting the complex humanitarian crisis in the DRC were perceived as disingenuous [20]. In this broader historical context, civil war and ongoing unrest led to a “toxic mix” [39] of chronic mistrust and sense of abandonment, following years of internecine rebel attacks, in which the national government has been ineffectual in restoring order. Some extreme views arose in our FGDs (“EVD is a creation come to exterminate the population”), a theory also promulgated by a local politician [40]. Thus, it appears that for some in the DRC, as in Guinea, EVD transformed existing grievances into a visceral fear of actual genocide [8]. Thus, conflict with outside agents can be understood from this backdrop of suspicion, aggravated by panic due to the rapid and lethal nature of the epidemic.

Our study is subject to several limitations. Survey questionnaire items were adapted from prior studies [5, 20]; however, the robust validation of the instrument in diverse contexts has not been performed. We translated FGDs from the original language which may have resulted in some intended nuances being lost. Because of the need to conduct the survey as the epidemic evolves, the comprehension of survey questions in French or other languages was not evaluated. Furthermore, for survey participants who did not speak French, questions and responses were translated into their preferred language, such that variations in interpretation may have been introduced. Social desirability bias may have influenced some participants’ responses, which tended to align with official recommendations. We did not examine the relation between knowledge, attitudes, and exposure to health promotion interventions or messages. Finally, the sample was a convenience sample purposively drawn from the area of the outbreak, with an over-representation of students and higher education level, but it would be of interest to assess attitudes toward Ebola in other geographic, political, and cultural contexts through a purposive sampling strategy.

Several recommendations emerge from our study that may inform current and future EVD responses. First, international agencies should aspire to a more sustained and wholistic approach to public health issues that matter to the community. The intense, vertical, and focused EVD response during this epidemic has not been well accepted in part because the community is unaccustomed to external aid and suspicious of disingenuous motives. This might serve to build a foundation of trust upon which foreign assistance in an epidemic or crisis situation could build. Second, strong communication and dialogue with community members in the local language should be foundational; language barrier was highlighted as a failing of the EVD response among FGD participants. Third, engaging trusted local experts (e.g., physicians and nurses) for public health messaging and case management should receive more emphasis. The response was characterized by a belief that local physicians and nurses “lacked the capacity” to deal with an EVD outbreak. Rather than build local capacity, the emphasis was on emergency containment, which ultimately became counter-productive as communities resisted the “top-down” control effort. Finally, real-time data collection, capturing emergent, on-the-ground, local barriers and facilitators [22] should be used to integrate community attitudes, behaviors, and responses into epidemiological research [41]. Our mixed-method study is a model that could be followed in future epidemics to complement biomedical approaches toward an effective outbreak response.

Anthropologic research during other EVD epidemics suggests that root causes of resistance or reticence relate to several domains, including rumours, fear, mistrust and lack of confidence in the authorities, and denial of the biomedical discourse [8, 11, 42]. Our findings support these themes, and further suggest that mistrust and lack of confidence in authorities have emerged as dominant factors in the current epidemic. These observations suggest that community engagement to halt this and future epidemics may require more than education and correcting misinformation, as previous authors have argued [43, 44]. A much more costly and complicated, yet genuine, commitment to the health of the Congolese people, might mean ending the hidden war [45] and not just stepping in when contagion threatens to spill across the borders.

## Supporting information

S1 File **Table A**. **Translation of quotations from French (original) to English**. Quotations are presented in the same order that they appear in the Results Section. **Table B**. **Correlation between questionnaire items related to dissatisfaction with Ebola response and/or mistrust of the Ebola response team**. Agreement with all statements was quantified using an ordinal Likert scale from 1 to 5. **Table C**. **Correlation between questionnaire items related to intentions to comply with Ebola control measures in case of Ebola illness or death in a family member**.
(DOCX)
