# Supplementary material for: Social resistance drives persistent transmission of Ebola virus disease in Eastern Democratic Republic of Congo: A mixed-methods study
Source: PLoS One. 2019 Sep 26;14(9):e0223104. doi: 10.1371/journal.pone.0223104 (PMC6762146; doi:10.1371/journal.pone.0223104)
Supplement: S1 File — Table A. Translation of quotations from French (original) to English. Quotations are presented in the same order that they appear in the Results Section. Table B. Correlation between questionnaire items related to dissatisfaction with Ebola response and/or mistrust of the Ebola response team. Agreement with all statements was quantified using an ordinal Likert scale from 1 to 5. Table C. Correlation between questionnaire items related to intentions to comply with Ebola control measures in case of Ebola illness or death in a family member. (DOCX) [file pone.0223104.s001.docx]

**Table A. Translation of quotations from French (original) to English.** Quotations are presented in the same order that they appear in the Results Section

| **Quotation in French** | **English translation** |
| --- | --- |
| L’épidémie est léthale et l’éclosion est forte, sans la participation de l’extérieur la maladie aurait déjà pris une autre dimension et peut-être meme franchir certaines frontières des pays limitrophes. Nous disons donc merci a tous ceux qui nous soutiennent. | “The epidemic is lethal and the eruption is strong, without the participation of the outside the sickness would have already taken another dimension and even crossed certain borders of neighboring countries. We therefore say thank you to all those who are supporting us.” (doctor, ID#11) |
| Il y a quelques jours, un poste de santé a été attaqué par la population en colère et deux policiers en sont sortis avec des lésions dues au jet de pierre. Il y a eu des coups de balles avec du dégât matériel. | “A few days ago, a health center was attacked by the angry community and two police officers were left with injuries due to throwing stones. There were gunshots and property damage.” (doctor, ID#16) |
| La population digère mal le nombre de morts élevé au niveau du CTE. Le CTE bien gardé, la population s’attaque au poste de santé qui a transféré le malade au CTE. | “The community is having a hard time digesting the high number of deaths at the ETC [EVD treatment centre]. The ETC is well guarded, so the community attacked the health post that transferred the patient to the ETC.” (doctor, ID#16) |
| Chacun a un leader à qui il fait confiance. Ces “étrangers au milieu” à cause de l’argent n’ont pas pris cet aspect en considération. | “Everyone has a leader that he has confidence in. These *étrangers au milieu* [strangers in our midst] because of money didn’t take this aspect into consideration.” (doctor, ID#17) |
| La formation devait se faire de bouche à l’oreille de façon informelle. | “The training should have been done *de bouche à l’oreille* [from mouth to the ear] in an informal manner.” (doctor, ID#18) |
| Un béninois va faire sa sensibilisation en français, un kinois en lingala. Et cela, vous pensez qu’il aura quel impact? | “A Béninois will do his messaging in French, a *Kinois* [person from country capital Kinshasa] in Lingala. And that, what impact do you think it will have?” (doctor, ID#17, note that the locally spoken languages are Swahili and Kinande, not French or Lingala) |
| C’est aussi un problème de culture. Non, vraiment la façon de gérer le corps c’est horrible. Notre culture ne tolère pas ça. Le corps est considéré comme une chose, un objet. | “It’s also a problem of culture. No, really, the way of handling the body is horrible. Our culture doesn’t tolerate this. The body is considered like a thing, an object.” (nurse and nun, ID#19) |
| Le corps … est emballé dans une bâche. Personne de la famille ne voit le corps. Notre culture veut que l’on dise au revoir à celui qui nous précède dans l’au-delà. C’est un moment fort pour nous. | “The body… is wrapped in a tarp. No one from the family sees the body. Our culture demands that we say goodbye to the one that goes ahead of us to the beyond. It’s a powerful moment for us.” (nurse and nun, ID#19) |
| une morgue de fortune en caoutchouc. | “*une morgue de fortune en caoutchouc* [a makeshift plastic morgue].” (doctor, ID#18, describing the way bodies are wrapped in tarpaulin sheets for safe burial) |
| Un proche tombe malade, il consulte au CTE, c’est tout. Vous ne le verrez plus ou mieux disons c’est une séparation sans possibilité de se revoir. | “A loved one falls ill, he goes to the ETC, that’s it. You will never see him again or better let’s say it’s a separation without possibility of seeing each other again.” (nurse and nun, ID#19) |
| Si le malade est un peu fort, il peut s’assoir en dehors de sa tente et sera vu par ses familiers bien sur en respectant une certaine distance de sécurité. | “If the patient has a little bit of strength, he can sit outside his tent and can be seen by his family, of course keeping a certain safe distance.” (doctor, ID#18) |
| On ne sait pas garder un corps plus de 24h. Et donc si contact avec familiers non possible, on l’enterre. | “We can’t keep the body more than 24 hours. So, if we can’t contact the family, we bury it.” (doctor, ID#18) |
| Un deuil sans dire au revoir au défunt!! C’est une malédiction ça. | “A funeral without saying goodbye to the deceased!! That is a curse, that is.” (community member, ID#14) |
| En cas de décès, la famille informée n’est pas convaincue qu’il s’agit de lui. | “In case of death, the family once informed is not convinced that it’s him.” (nurse and nun, ID#19) |
| Le problème du repas se posait avant mais maintenant, pas vraiment parce que la restauration a été confiée désormais aux religieuses. Dans le temps, cette responsabilité était confiée à un restaurant qui amenait le repas à des heures irrégulières. | “There was a problem with meals earlier, but now not really, because the catering has been delegated from now on to the nuns [religious order]. Previously, this responsibility was given to a restaurant that brought meals at irregular times.” (nurse and nun, ID#19) |
| Les sœurs donnent le repas oui mais quel genre de repas pour quel genre d’intestin. Certains malades ont des intestins fragiles, mais mangent la même nourriture que les autres. | “the nuns give the meals, yes, but what kind of meal for what kind of intestine? Some of the sick have fragile intestines, but eat the same food as others.” (doctor, ID#18) |
| Les médicaments ne sont pas suffisants. Nous avons toujours peur parce que le processus est long pour que ces visiteurs là puissent croire à ce que tu leur dis. Ils croient pas aux gens. | “The medications are not sufficient. We are still scared because the process is slow for these visitors here to be able to believe in what you are telling them. They don’t believe people.” (community member, ID#05) |
| Tout le monde veut en avoir [vaccin]. Et selon les critères, seulement ceux qui ont été en contact avec les suspects. Et c’est une procedure pour vérifier la véracite des propos d’un demandeur de vaccin. | “Everyone wants [the vaccine]. And according to the criteria, only those who were in contact with the suspected cases. And it’s a procedure to verify the veracity of the claims of someone who is asking for the vaccine.” (doctor, ID#01) |
| Nous informons la hiérarchie sanitaire de [nom du village]… mais personne ne décroche. L’équipe finalement informée nous dit qu’elle saurait faire le déplacement que le jour suivant. A cause de l’insécurité, pas de sortie nocturne. Le corps reste au centre de santé sans morgue équipée. | “We informed the health authorities of [name of town]… but no one answered the phone. Finally, having informed the team, they told us that they would come the next day. Because of the security risk, no travel at night. The body stayed at the health centre without an equipped morgue.” (doctor, ID#11) |
| [ONG] fait une médecine des troupeaux. Quand un mouton est malade, et bien il faut soigner tous les moutons de la même façon. Ce n’est pas une médecine individualisée prise cas par cas, non. | “[NGO] is doing *médecine des troupeaux* [herd medicine]. When a sheep is sick, well, you have to treat all the sheep in the same way. It’s not individualized medicine on a case-by-case basis, no.” (doctor, ID#18, in this and subsequent quotations, NGO was used in place of the named international organization named by the FGD participant) |
| [ONG] a un protocole figé. Malade Ebola égale ce protocole. | “[NGO] has a *protocole figé* [rigid frozen protocol]. EVD patient equals protocol…” (doctor, ID#18) |
| Pour [ONG], objectif égale limiter épidémie, isoler le malade (même s’il meurt, pas de problème, pourvu qu’il ne contamine pas les autres). | “For [NGO], the objective is to limit the epidemic, isolate the sick. Even if he dies, no problem, so long as he doesn’t contaminate others.” (doctor, ID#17) |
| Comprendre ce qui s’est passé là c’est difficile. Moi ici, j’ai démissionné juste une semaine après. Je suis recruté. J’arrive sur [nom du village], je ne sais même plus à qui m’adresser. Celui-ci vous dit va là et celui-la vous dit l’autre. Bref, on commence. Là je me loge. Je me nourris. On me dit, vous devez être à [nom du village] (30 km de [nom du village] où je restais) à 6h du matin. Et voilà le matin pas de moyen de déplacement. Je prends une moto. Et le soir idem… Et voilà je suis parti. Les gens sont démotivés… Et si les locaux se désengagent du processus, mais ça sera une catastrophe humanitaire grave. | “Understanding what is going on is difficult. Me, I quit just a week into it. I was recruited. I arrived in [city name], I didn’t even know whom to speak to. One person says go there, and another says something else. Finally, we start. Now I pay for my own accommodation. I pay for my own food. They tell me, you have to be in [name of town] (30 km away from [name of town] where I was staying) at 6am in the morning. And then in the morning there was no means of transportation. I take a motorcycle, and the evening, the same thing… So look, I left. People are de-motivated… And if the local people disengage from the process, well it will be a serious humanitarian catastrophe.” (doctor, ID#18, geographic names have been removed to preserve anonymity) |
| On sait lire l’esprit des kinois, comme qui dirait no Ebola no job. Les kinois sont venus très nombreux, ils ont peur du risque et leur barème haut par rapport à celui des locaux qui en plus se sacrifient pour la population. Les locaux sont mal payés raison de beaucoup de démissions. Avez-vous déjà appris qu’il y a un kinois qui a démissionné? Les disparités sont criantes et nous avions même écrit un memo a la hiérarchie. Si certains locaux restent encore c’est par patriotisme, un bénévolat. | “We can read the spirit of the *Kinois* [workers from Kinshasa], which is ‘no EVD, no job.’ The *Kinois* have come in large numbers, they are afraid of the risk and their remuneration is high relative to that of the locals who in addition are sacrificing themselves for the community. The locals are poorly paid, and that’s the reason for a lot of people quitting. Have you ever heard of a *Kinois* who quit? The disparities are striking and we’ve even written a memo to the authorities. If some locals remain, it’s out of patriotism, a volunteer spirit.” (doctor, ID#17) |
| … malheureusement sans prime de risqué. Le gouvernement devrait se pencher sur leur cas. | “…[despite deaths and infections], unfortunately without hazard pay. The government should think about their case.” (doctor, ID#01) |
| Et nos recettes ne dependent que des consultations et activités payées par les malades. . Voila, vous comprenez... Tous ces facteurs là font que les recettes baissent. | “Our revenues depend solely on consultation [fees] and activities paid by the patients. There, you understand...all these factors mean that the revenues go down.” (doctor, ID#01) |
| Quant au personnel soignant sans prime de risqué, c’est la méfiance totale envers des cas comme ceux là. | “As for the health personnel without hazard pay, it’s total fear around cases like these.” (doctor, ID#11) |
| Le message que donnent les politiciens qu’Ebola est une création pour venir exterminer la population de [nom du village]. Les gens sont manipulés et s’en tiennent à ça. | “The message that the politicians are giving is that EVD is a creation come to exterminate the population of [name of town]. People are manipulated and hold to this.” (doctor, ID#18) |
| par exemple, on refuse le vaccin aux femmes enceintes alors que nous savons que les femmes enceintes sont toujours vaccinées pour protéger les bébés. On se demande si cette politique n’est pas une façon de nous exterminer. | “for example, they are refusing the vaccine to pregnant women, yet we know that pregnant women are always vaccinated to protect the babies. We are asking if this policy is not a way to exterminate us.” (IDP, ID#10) |
| Nous nous sommes aidés mutuellement. Dire vrai, il y avait plus malade que moi, très affaibli…. Nous avons soigné les malades dans le CTE. Personnellement j’ai fait le bain de malade d’Ebola comme moi. Il s’agit d’un collègue. Vider son pot. Il n’avait aucune force. Nous avons reconnecté des perfusions aux malades dépassés psychologiquement qui déconnectaient les leurs. … On communiquait avec nos collègues qui sont dehors par téléphone. Nous passions les besoins en médicaments (réquisition) au près d’eux et ils nous les jetaient. Nous avons aidé beaucoup de malades devant ce protocole [ONG] qui ne s’adapte pas au cas par cas. | “We helped each other out, mutually. Truth be told, there were others sicker than I was, very weakened…We treated the sick in the ETC. Personally, I bathed another patient who had EVD like me. It was a colleague. Emptying his chamberpot. He had no strength. We reconnected the drips of patients who were psychologically overcome who had disconnected theirs…We communicated with our colleagues who were outside by telephone. We told them the medication needs (requests) and they would throw them to us. We helped a lot of sick people in the face of this [NGO] protocol that doesn’t adapt to the case-by-case.” (nurse, ID#20) |

**Table B. Correlation between questionnaire items related to dissatisfaction with Ebola response and/or mistrust of the Ebola response team**. Agreement with all statements was quantified using an ordinal Likert scale from 1 to 5.

|  | Statement 1 | Statement 2 | Statement 3 | Statement 4 | Statement 5 |
| --- | --- | --- | --- | --- | --- |
| Statement 1 |  |  |  |  |  |
| Statement 2 | ρ=0.68  p<0.0001 |  |  |  |  |
| Statement 3 | ρ=0.25  p<0.0001 | ρ=0.29  p<0.0001 |  |  |  |
| Statement 4 | ρ=0.35  p<0.0001 | ρ=0.29  p<0.0001 | ρ=0.33  p<0.0001 |  |  |
| Statement 5 | ρ=0.32  p<0.0001 | ρ=0.31  p<0.0001 | ρ=0.27  p<0.0001 | ρ=0.54  p<0.0001 |  |

Statement 1: “I am not satisfied with the Ebola response in Eastern DRC.”

Statement 2: “The Ebola response is not well organized.”

Statement 3: “The foreigners come to assist with Ebola control have a poor understanding of the local culture and regional situation.”

Statement 4: “I don’t trust the foreigners come to assist with Ebola control.”

Statement 5: “The foreigners have come to make a profit from this epidemic.”

**Table C. Correlation between questionnaire items related to intentions to comply with Ebola control measures in case of Ebola illness or death in a family member.**

|  | Intention 1 | Intention 2 | Intention 3 | Intention 4 |
| --- | --- | --- | --- | --- |
| Intention 1 |  |  |  |  |
| Intention 2 | ρ=0.46  p<0.0001 |  |  |  |
| Intention 3 | ρ=0.38  p<0.0001 | ρ=0.54  p<0.0001 |  |  |
| Intention 4 | ρ=0.36  p<0.0001 | ρ=0.50  p<0.0001 | ρ=0.45  p<0.0001 |  |

Intention 1. If a family member was infected with Ebola, would you bring him to the treatment centre? (“no” response considered marker of resistance)

Intention 2. If a family member was infected with Ebola, would you hide him/her from the health authorities? (“yes” response considered marker of resistance)

Intention 3. If a family member died at home with suspected Ebola, would you touch the body? (“yes” response considered marker of resistance)

Intention 4. If a family member died at home with suspected Ebola, would you accept an official burial team take care of the body? (“no” response considered marker of resistance)
